# Chronic rhinosinusitis with nasal polyps and allergic rhinitis as different multimorbid treatable traits in asthma

**DOI:** 10.1016/j.jacig.2023.100134

**Published:** 2023-07-03

**Authors:** José Antonio Castillo, Vicente Plaza, Gustavo Rodrigo, Berta Juliá, César Picado, Cristina Fernández, Joaquim Mullol

**Affiliations:** a Pneumology Department, Hospital Universitari Dexeus, Barcelona, Spain; b CIBER of Respiratory Diseases, Spain; c Group of Rhinitis, Rhinosinusitis, and Nasal Polyps, Area of Asthma, SEPAR, Spain; d Pneumology Department, Hospital Santa Creu i Sant Pau, Barcelona, Spain; e Emergency Departament, Hospital Central de las Fuerzas Armadas, Montevideo, Uruguay; f Medical Department, MSD Madrid, Spain; g Pneumology Department, Hospital Clinic, Universitat de Barcelona, Barcelona, Spain; h Preventive Medicine Department, Complejo Hospitalario Universitario de Santiago de Compostela, Instituto de Investigación Sanitaria Santiago de Compostela, Fundación IMAS, Santiago de Compostela, Spain; i Clinical and Experimental Respiratory Immunoallergy, IDIBAPS & Rhinology Unite and Smell Clinic, ENT Department, Hospital Clínic Barcelona, Universitat de Barcelona, Spain

**Keywords:** Asthma, allergic rhinitis, chronic rhinosinusitis with nasal polyps, united airway disease, asthma severity, asthma control

## Abstract

**Background:**

Respiratory multimorbidities are linked to asthma, such as allergic rhinitis (AR) with early allergic asthma and chronic rhinosinusitis (CRS) with nasal polyps (CRSwNP) with late nonallergic asthma.

**Objective:**

Our aim was to investigate the association of asthma severity and control with specific upper airway phenotypes.

**Method:**

Patients with asthma were prospectively recruited from 23 pulmonology and ear, nose, and throat clinics. Asthma severity and control, as well as upper airway comorbidities (AR and non-AR [NAR], CRSwNP, and CRS without nasal polyps [CRSsNP]) were assessed according to international consensus guidelines definitions.

**Results:**

A total of 492 asthmatic patients were included. Half of the asthmatic patients (49.6%) had associated rhinitis (37.0% had AR and 12.6% had NAR) and 36.2% had CRS (16.7% had CRSsNP and 19.5% had CRSwNP), whereas 14.2% had no sinonasal symptoms. Most cases of AR (78%) and NAR (84%) were present in patients with mild-to-moderate asthma, whereas CRSwNP was more frequent in patients with severe asthma (35% [*P* < .001]), mainly nonatopic asthma (44% [*P* < .001]). Patients with severe asthma with CRSwNP had worse asthma control, which was correlated (*r* = 0.249 [*P* = .034]) with sinus occupancy. Multiple logistic regression analysis showed that late-onset asthma, intolerance of aspirin and/or nonsteroidal anti-inflammatory drugs, and CRSwNP were independently associated with severe asthma.

**Conclusion:**

Severe asthma is associated with CRSwNP, with sinus occupancy affecting asthma control. This study has identified 2 main different upper airway treatable traits, AR and CRSwNP, which need further evaluation to improve management and control of patients with asthma.

Asthma is a heterogeneous condition with many different subphenotpyes with differences in severity, natural history, multimorbidity, and response to treatment**.** Although most patients with asthma can be controlled with optimal therapy,^1^ a number of asthmatic patients show severe uncontrolled asthma.^1^^,^^2^ These patients are at risk for increased burden, such as frequent and severe exacerbations, and they account for a significant amount of health care costs.^1^^,^^3^

The synergic and concurrent interactions between upper and lower airways is now well recognized, thus supporting use of the term *united* (also known as "unified airway disease"^4^ or “1 airway, 1 disease”). Rhinitis and chronic rhinosinusitis (CRS) constitute common multimorbidities with asthma, influencing the disease progression from nose to bronchi.^5^ There appears to be a clear correlation between asthma severity and control and the presence and severity of rhinitis or CRS,^6^ this being more evident in CRS with nasal polyps (CRSwNP). Airway inflammation in severe chronic upper airway diseases is likely to contribute to the worsening of asthma severity and control.^7^^,^^8^

To better understand the origin and evolution of asthma, especially in cases of severe asthma, there is a need to characterize asthma^9^^,^^10^ and CRS^11^ phenotypes and endotypes. Important clinical features in phenotyping asthma^12^^,^^13^ are age at disease onset, presence of concomitant allergy, and frequency of exacerbations and airflow limitation. Several other clinical phenotypes and coexisting conditions, such as sex, smoking, and obesity,^14^ have also been described. In addition, aspirin-exacerbated respiratory disease (AERD) or nonsteroidal anti-inflammatory drug (NSAID)-exacerbated respiratory disease^15^^,^^16^ (N-ERD) has been analyzed.^17^^,^^18^ Recent “cluster” analysis, based on inflammatory cells (eosinophils and neutrophils) and biomarkers (IgE and IL-5) in sputum from patients with asthma or integration of the transcriptomic^19^^,^^20^ signatures into the analysis^21^^,^^22^ and nasal secretions from CRSwNP,^23^ have identified a number of potential endotypes to study upper and lower airway multimorbidity. Recently, patients with asthma have been broadly split into 2 distinct endotypes—type 2 asthma and non–type 2 asthma—depending on the degree of type 2 inflammation. The fact that patients with asthma can present with many clinical phenoptypes has justified the need for a precision medicine strategy based on the presence of treatable traits.

In this direction and from a clinical point of view, involvement of upper airway disease should also be considered in every patient with asthma in the light of a precision medicine approach, as it has been identified as a treatable trait that needs to be assessed and treated to improve outcome.^24^

The aim of this study was to investigate, first, the prevalence of sinonasal diseases such as rhinitis (allergic or nonallergic) and CRS (CRSwNP and CRS without nasal polyps [CRSsNP]) in patients with asthma, and second, the phenotype characteristics that are significantly associated with asthma severity and control.

## Methods

### Ethics approval

This study was registered at ClinicalTrials.gov under the identifier NCT01513837 and given the title “IRIS-Asthma” (an acronym for Prevalence, Severity, and Impact of Rhinitis and Rhinosinusitis by Asthma Severity and Control). The study was approved by the Hospital Santa Creu i Sant Pau ethics committee, and all subjects provided written informed consent.

### Study population

Adult patients (aged 18-70 years) who had a physician´s diagnosis of asthma (according to the Global Iniciative for Asthma [GINA]^25^ and the Spanish guideline for asthma management^26^) that had been evolving for at least 1 year and who had visited outpatients clinics at 23 centers (19 in Spain and 4 in Latin America) between 2012 and 2013 were consecutively screened and selected for the participation in this study. Patients with nonrelated major comorbidities (eg. emphysema, congestive heart failure, and anemia) and pregnancy were excluded. Current smokers and former smokers (>10 pack years) were allowed for inclusion only if the patient had at least a 12% improvement in FEV_1_ value and FEV_1_ percent predicted after inhalation of 400 μg of salbutamol, as well as a normal diffusion capacity of greater than 90% of the predicted value. All patients had to have had stable disease for at least 4 weeks before entering the study.

### Study design

In this cross-sectional multicenter study with a prospective protocol, all patients attended 1 visit at which clinical and questionnaire characterization was undertaken.

#### Asthma characteristic severity and control

Consecutive asthmatic patients attending pulmonologist and allergy outpatient clinics using a multidisciplinary approach with ENT specialists were included. Asthma was diagnosed on the basis of clinical history and lung function. Asthma severity was stratified according to GINA^25^ and control was stratified according to Asthma Control Test score.^27^ Number of exacerbations was defined as the number of prednisone bursts needed to control increased asthma symptoms in the past 12 months.

#### Lung function

Spirometry with a bronchodilator test was performed according to the European Respiratory Society recommendations.^1^ FEV_1_ and forced vital capacity values were obtained from flow-volume curves and expressed as absolute values, percentage of predicted normal values, and ratio of FEV_1_ value to forced vital capacity (Tiffeneau index).

#### Atopic status

Atopic status was assessed on the basis of serum-specific and total IgE levels (RAST and CAP) in response to a panel of common aeroallergens by means of Inmuno-CAP (Phadia, Uppsala, Sweden) or a positive result of a skin prick test (SPT) to common aeroallergens. Sensitization to the most common aeroallergens was tested according to SPT practical guide in allergy.^29^

#### Inflammatory status

Inflammatory status was assessed through exhaled nitric oxide (Feno) levels and blood count of peripheral eosinophils. Feno level measured with a Niox Mino (Model 03-1100; Aerocrine, Solna, Sweden) was preferentially used. Intolerance of aspirin (AIA) or NSAIDs was assessed by clinical history and/or aspirin challenge.

#### Sinonasal phenotypes

Allergic rhinitis (AR) and non-AR (NAR) were diagnosed according to the Allergic Rhinitis and Its Impact on Asthma (ARIA)^28^ definitions by using nasal symptoms and a positive result of an SPT to clinically relevant aeroallergens.^29^ AR severity was rated according to the m-ARIA severity classification.^30^

CRSwNP and CRSsNP were diagnosed according to the European Position Paper on Rhinosinusitis and Nasal Polyps.^31^ Symptoms for AR and CRS were self-evaluated separately by using Visual Analog Scale (VAS) score (0-100 mm). Loss of smell (LoS) was classified as normal or normosmia (VAS score 0-10 mm), partial loss or hyposmia (VAS score > 10-70 mm), or total loss or anosmia (VAS score > 70 mm).

Nasal endoscopy and sinonasal computed tomography (CT) were also performed, and the results were categorized according to international scores.^32^

### Analysis

#### Sample size

Approximately 450 patients with asthma needed to be enrolled in the study. This number was achieved by assuming the participation of 18 centers, with each of them recruiting 25 patients on average and losses somewhat higher than 10%. To estimate the precision provided by such a number, a 50% rhinitis/rhinosinusitis comorbidity rate was assumed. On the basis of these data, precision would be less than 5% (very close to 4.6%). If another prevalence rate (probably more realistic) for rhinitis/rhinosinusitis comorbidity were assumed (eg, a rate close to 80%), the precision would be on the order of 3.7%. In addition, because losses have been overestimated, the total number of patients to be recruited for the study appears more than adequate to answer the research question.

#### Statistical analysis

The number of available subjects (N= 492) was greater than that required according to sample size calculations from the total of 23 centers. Data values are given as means with SDs, medians (interquartile ranges [IQR]), or percentages. The means of the quantitative outcomes tested were compared between groups by using ANOVA or a median test, whereas a chi-square test was used for qualitative outcomes. In the case of quantitative outcomes, their association with the Pearson correlation coefficient was evaluated. Multivariate logistic regression was used to estimate an association of independent outcomes. We included those that were significant (*P* < .05) in the univariate analysis. Complete hierarchic models were estimated, and confusion was assessed by comparing the change in effect greater than 10%. Adjusted odds ratios (ORs) and their 95% CI are presented. *P* values less than .05 were considered statistically significant. SPSS software, version 16.0 (2008) (SPSS for Windows, Chicago, Ill) was used for all statistical analyses.

## Results

### Population and demographic characteristics

In all, 492 patients with asthma, mean age 46 (33-58) years old, 70.5% being females, who met the inclusion and exclusion criteria participated in this study. Two patients were excluded for lack of essential demographic data. The characteristics of the study population by descriptive analysis of demographics, clinical, and functional characteristics, are shown in Table I.

**Table I:** Demographic, clinical, and functional characteristics of patients with asthma according to the GINA severity classification

Asthma characteristic	Global		Intermittent		Mild persistent		Moderate persistent		Severe persistent		*P* value
Severity, no. (%)	492 (100)		86 (17.3)		121 (24.6)		154 (31.4)		131 (26.7)		
Age (y), median (25-75 IQR)	46	(33-58)	39	(28-53)	42	(29-56)	45	(35-57)	51	(41-61)∗∗∗	<.001
Time since asthma onset (y), median (25-75 IQR)	13	(5-25)	11	(4-19)	11	(3-21)	12	(5-25)	20	(10-31)∗∗∗	<.001
BMI (kg/m^2^), median (25-75 IQR)	26	(23-30)	26	(23-28)	25	(23-30)	26	(24-30)	27	(24-30)∗	.142
Female sex, no. (%)	347	(70.5)	57	(66.3)	89	(73.6)	108	(70.1)	93	(71.0)	.728
Sinonasal comorbidity, no. (%)											
None	70	(14.2)	10	(11.6)	24	(19.8)	21	(13.6)	15	(11.5)	<.001
NAR	62	(12.6)	14	(16.3)	17	(14.0)	21	(13.6)	10	(7.6)	—
AR	182	(37.0)	35	(40.7)	45	(37.2)	62	(40.3)	40	(30.5)	—
CRSsNP	82	16.7	16	(18.6)	24	(19.8)	22	(14.3)	20	(15.3)	—
CRSwNP	96	19.5	11	(12.8)	11	(9.1)	28	(18.2)	46	(35.1)∗∗∗	—
ACT score (pts), median (25-75 IQR)	21	(16-24)	23	(20-25)	22	(18-25)	20	(17-23)	17	(12-21)∗∗∗	<.001
Asthma exacerbations, no. (%)											
0	310	(63.0)	73	(84.9)	95	(78.5)	91	(59.1)	51	(38.9)	<.001
1-3	165	(33.5)	12	(14.0)	24	(19.8)	58	(37.7)	71	(54.2)∗∗∗	—
≥4	17	(3.5)	1	(1.2)	2	(1.7)	5	(3.2)	9	(6.9)	—
Sinonasal CT score (pts), median (25-75 IQR)	4	(0-11)	4	(0-9)	2	(0-5)	4	(0-12)	8	(1-15)∗	.014
LoSss of smell (VAS score), no. (%)											
Normosmia	167	(43.6)	36	(51.4)	45	(53.6)	54	(43.9)	32	(30.2)	.001
Hyposmia (10-70 mm)	159	(41.5)	28	(40.0)	30	(35.7)	55	(44.7)	46	(43.4)	—
Anosmia (>70 mm)	57	(14.9)	6	(8.6)	9	(10.7)	14	(11.4)	28	(26.4)∗∗∗	—
Blood eosinophil count (cell/μL), median (25-75 IQR)	250	(112-415)	225	(126-400)	230	(117-428)	250	(100-400)	300	(110-590)	.024
Feno level (ppb), median (25-75 IQR)	26	(15-45)	24	(14-47)	27	(17-43)	24	(15-44)	30	(16-46)	.495
FEV_1_ (%), median (25-75 IQR)	91	(75-103)	102	(91-110)	95	(88-108)	90	(75-100)	65	(53-86)∗∗∗	<.001
IgE (IU/mL), median (25-75 IQR)	150	(56-377)	125	(29-276)	124	(47-311)	130	(61-293)	193	(77-539)∗	.088
Oral steroid intake, no. (%)	55	(11.2)	0	(0.0)	2	(1.7)	7	(4.5)	46	(35.1)∗∗∗	<.001
Positive SPT result, no. (%)	298	(71.1)	50	(70.4)	78	(76.5)	90	(69.2)	80	(69.0)	.588
AERD/N-ERD, no. (%)	72	(15.2)	3	(3.8)	17	(14.4)	21	(14.5)	31	(23.7)∗∗	.002
Smoking habit, no. (%)	47	(9.6)	12	(14.0)	13	(10.7)	18	(11.7)	4	(3.1)∗∗	.025
Packs/y, (25-75 IQR)	5	(2-20)	9	(3-28)	2	(1-9)	7	(2-15)	6	(2-24)	.173

*ACT*, Asthma Control Test; *BMI*, body mass index; *pts*, points.

Severe versus nonsevere asthma.

∗ *P* < .05.

∗∗ *P* < .01.

∗∗∗ *P* < .001.

### Atopy

Most of the asthmatic patients (71.1%) showed a positive result of an SPT to common aeroallergens, with more patients with CRSsNP presenting a positive SPT result than did patients with CRSwNP (76.8% vs 67.8% [*P* < .001]) (see Table E1 in this article’s Online Repository at www.jaci-global.org).

### Inflammatory biomarkers

Only blood eosinophilia (but not Feno or total serum IgE levels) was higher in patients with severe asthma (Table I). Despite atopy, patients with asthma and CRSwNP showed higher blood eosinophilia (*P = .*024), Feno levels (*P* < .001), and total serum levels IgE (*P* < .001) than those with other sinonasal phenotypes (see Table E1 in this article’s Online Repository at www.jaci-global.org).

### NSAID sensitivity

The asthmatic patients with a history of AIA or AERD/N-ERD^22^^,^^23^ had a prevalence of 15% (72 of 473). AERD/N-ERD was strongly associated with severe asthma (OR = 7.85 [*P = .*001]) and with CRSwNP phenotype (OR = 9.05 [*P* < .001]) (Table I).

### Sinonasal phenotypes

**Fig 1:**
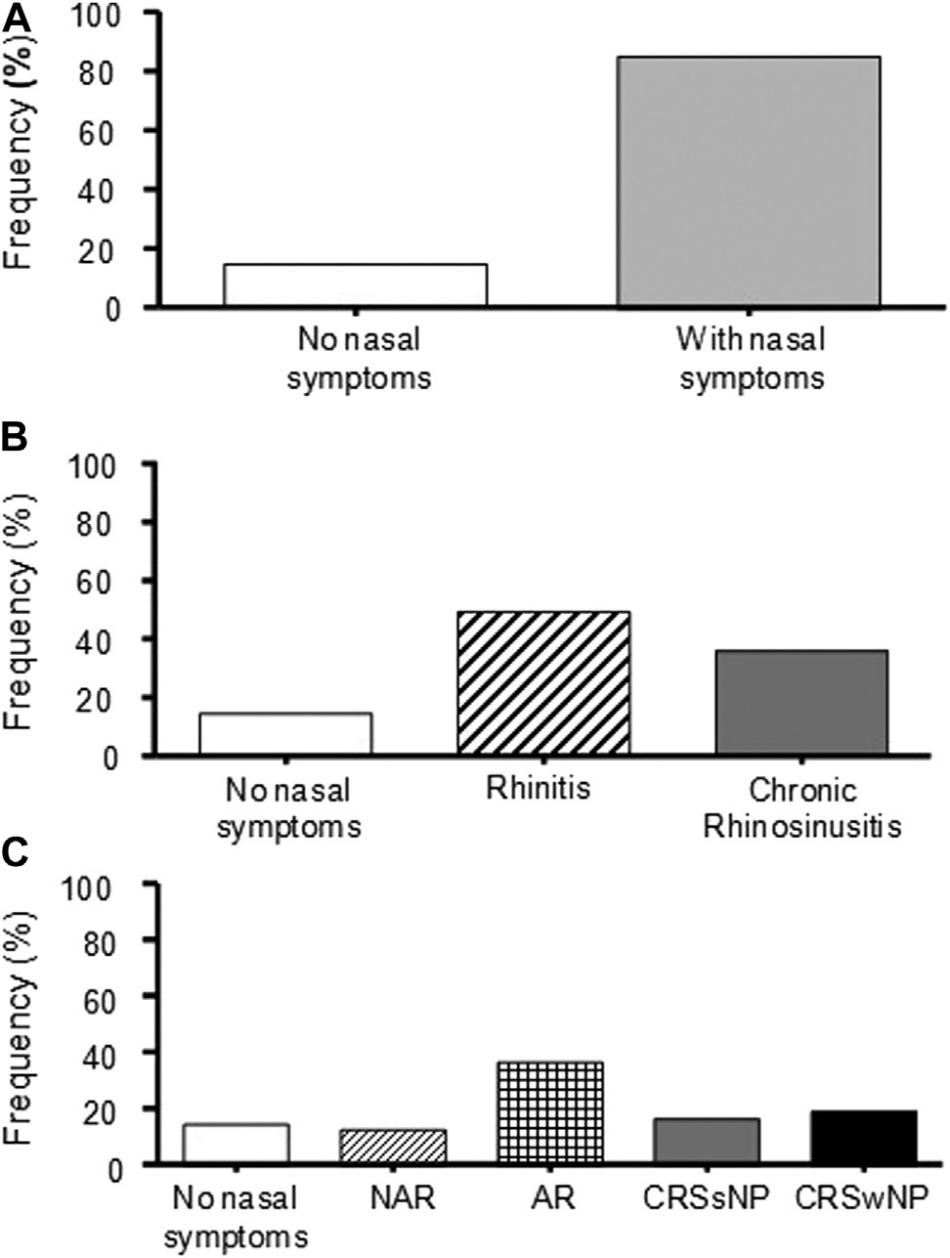
Frequency of sinonasal phenotypes in asthma (N = 492). Percentage of patients using nasal symptoms (86%) (**A**); using ARIA and European Position Paper on Rhinosinusitis and Nasal Polyps (EPOS) guideline definitions (50% had rhinitis whereas 36% had CRS) (**B**); and using allergy testing, nasal endoscopy, and sinus CT scan (14.2% were without sinonasal disease, 13% had NAR, 37% had AR, 16% had CRSsNP, and 20% had CRSwNP (**C**).

**Fig E1:**
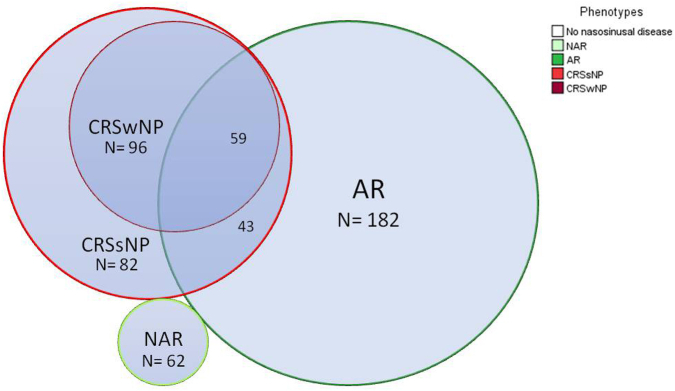
Venn diagram for sinonasal phenotypes in asthma (N = 492). According to ARIA and European Position Paper on Rhinosinusitis and Nasal Polyps (EPOS) guideline definitions. The diagram includes patients with rhinitis (n = 244 [50%]), patients with both AR (n = 182 [37%]) and NAR (n = 62 [12.6%]), patients with CRS (36%), patients with both CRSsNP (n = 82 [16.7%]) and CRSwNP (n = 96 [19.5%]), and a few patients without sinonasal disease (n = 70 [14.3%]).

Prevalence of rhinitis and CRS in asthma is shown in Fig 1 and Fig E1 (see this article’s Online Repository at www.jaci-global.org). Most of the asthmatic patients (86%) reported nasal symptoms and, according to the ARIA and European Position Paper on Rhinosinusitis and Nasal Polyps definitions, 49.6% of asthmatic patients had rhinitis whereas 36.2% had CRS. On the basis of a positive result of an SPT to clinically relevant aeroallergens, patients with asthma and rhinitis were classified as having AR (37.0%) or NAR (12.6%).

Finally, on the basis of nasal endoscopy and sinus computed tomography (CT) scan, patients with asthma and CRS were classified as having CRSwNP (19.5%) or CRSsNP (16.7%).

### Asthma severity and control

#### Asthma severity

According to the GINA asthma severity classification, the distribution of the study population was as follows: 17.3% had intermittent asthma and 82.7% had persistent asthma (24.6% mild, 31.4% moderate, and 26.7% severe). According to patients' Asthma Control Test score, 58.5% of cases were controlled, 20.3% were partially controlled, and 21.1% were uncontrolled. Body mass index increased with asthma severity. Patients with severe asthma (mean age 51 years) and those with CRSwNP (mean age 50 years) were among the oldest patients, whereas patients with intermittent asthma (mean age 39 years) and those with AR (mean age 43 years) were among the youngest. Time since onset of asthma was significantly higher in the case of patients with severe asthma (mean age 20 years) and patients with asthma and CRSwNP (mean age 16 years), (see Table E1 in this article’s Online Repository at www.jaci-global.org).

Asthma exacerbations, intake of oral steroids in the past 3 months, and lower FEV_1_ predicted values were predominantly also observed in patients with severe asthma and those with asthma and CRSwNP. The level of serum total IgE increased with asthma severity despite allergy condition and was highest in patients with severe asthma and CRSwNP.

#### Impact of rhinitis and CRS in asthma severity and control

**Fig 2:**
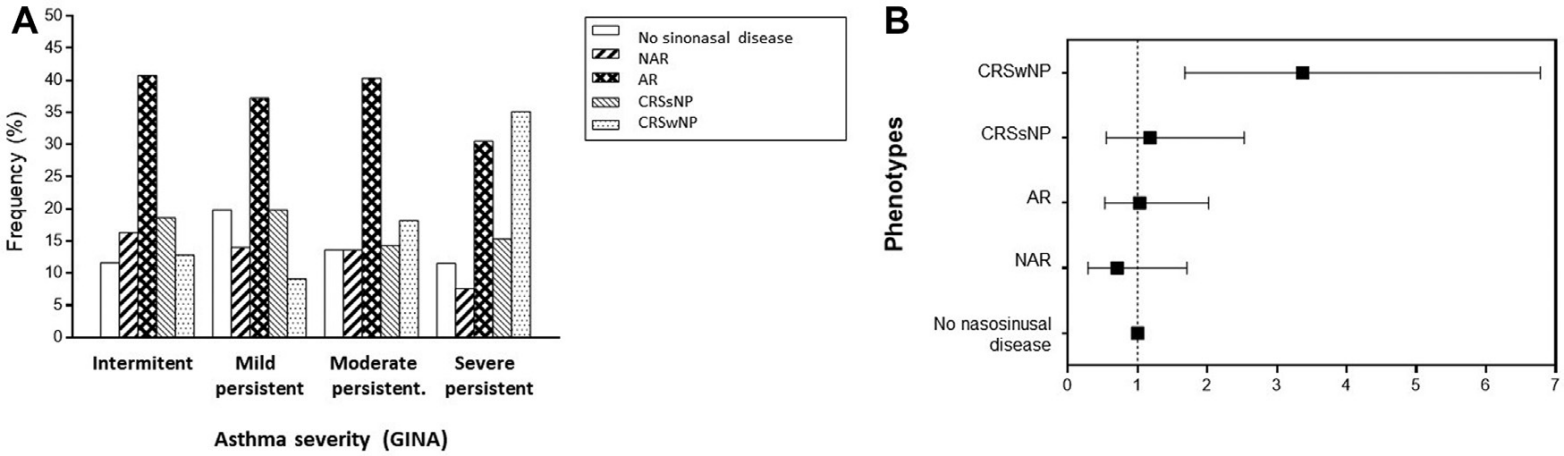
Frequency (**A**) and association (**B**) of sinonasal diseases according to asthma severity. CRSwNP shows an increased frequency of (35.1% [*P* < .001]) and association with (OR = 3.37 [95% CI = 1.69-6.78] [*P* < .001]) severe persistent asthma. The frequency and association of AR and NAR and CRSsNP are similar in all asthma severity groups. Frequency is expressed as a percentage, and association is expressed as an OR (95% CI).

Most patients with AR (78%) or NAR (84%) had intermittent or mild-to-moderate asthma. A similar frequency of CRSsNP (from 15% to 20%) was found in all levels of asthma severity, whereas CRSwNP was clearly associated with severe asthma (35% [*P* < .001]; OR = 3.4 [95% CI = 1.68-6.78]) (Fig 2). The prevalence of CRSwNP was significantly higher in patients with nonatopic asthma than in patients with severe atopic asthma (44% vs 32% [*P* < .01]).

LoS was present in 54.5% of asthmatic patients (hyposmia 41.5%, anosmia 14.9%). LoS severity was greater (22 mm [IQR = 0-75 (*P* < .001)]) and anosmia more frequent (26.4% [*P* < .001]) in patients with severe persistent asthma than in those with moderate (10 mm [IQR = 0-50]) (11.4%), mild (0 mm [IQR = 0-28]) (10.7%), or intermittent asthma (0 mm [IQR = 0-45]) (8.6%). In addition, LoS was more severe (38 mm [IQR = 2-76] vs 0 mm [IQR = 0-20], *P* < .001) and anosmia was more frequent (28.1% vs 3.9% [*P* < .001]) in patients with CRS than in those with rhinitis, and even more severe (50 mm [IQR = 11-89] vs 20 mm [IQR 0-56] [*P* < .001]) and anosmia more frequent (40.6% vs 13.4% [*P* < .001]) in patients with CRSwNP than in those with CRSsNP (see Table E1 in this article’s Online Repository at www.jaci-global.org).

Futhermore, LoS was more severe in patients with asthma and AERD/N-ERD than in those without asthma and AERD/N-ERD (33 mm [IQR = 2-81] vs 3 mm [IQR = 0-48]) [*P* < .001]).

**Fig 3:**
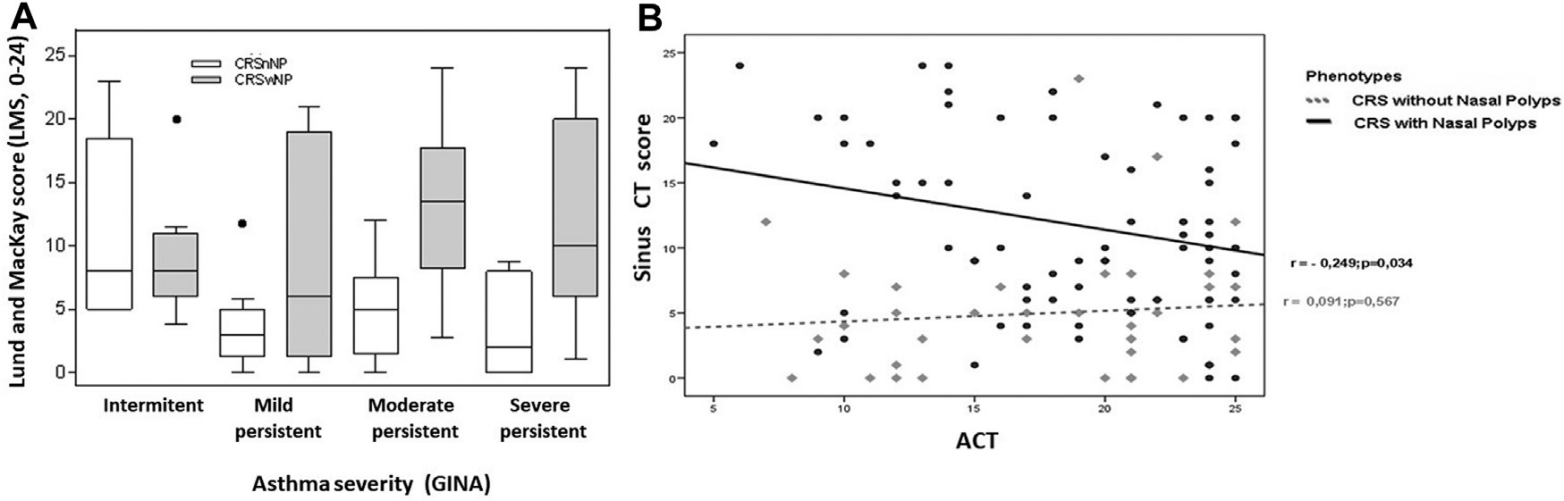
Sinonasal CT LMS scores in patients with CRSwNP and CRSsNP according to asthma severity. **A,** Patients with severe asthma shows higher LMS scores than patients with nonsevere asthma do (*P = .*014). **B,** LMS scores in patients with CRSwNP (*r* = –0.249; *P = .*034), but not in patients with CRSsNP (*r* = 0.091; *P = .*567), were negatively correlated with Asthma Control Test (ACT) score. The Pearson correlation coefficient (*r*) was used for the correlation. Quantitative variables are expressed as medians and IQRs.

Nasal endoscopy was performed in all of the patients, and sinus CT scan was performed in 181 asthmatic patients, mainly in those with CRS symptoms (CRSwNP [n = 73 (76%)], CRSsNP [n = 42 (51.2%)], AR [n = 43 (23%)], NAR [n = 19 (30.6%)], and those without sinonasal symptoms [n = 4 (5%)]), and according to asthma severity, in patients with intermittent (n = 25 [13.8%]), mild (n = 39 [21.5%]), moderate (n = 57 [31.5%]), and severe (n = 60 [33.1%]) persistent asthma. Lund-Mackay score (LMS), as determined by CT scan, was significantly higher in patients with severe asthma (8 [IQR = 1-15] [*P = .*014]) than in patients with intermittent (4 [IQR= 0-9]), mild (2 [IQR = 0-5]), and moderate (4 [IQR 0-12]) persistent asthma. LMS was also significantly higher in patients with CRSwNP (10 [IQR 6-18] [*P* < .001]) than in patients with CRSsNP (5 [IQR 1-7]) and moderate-to-severe asthma (Fig 3, *A*) and in patients with asthma and AERD/N-ERD than in those without AERD/N-ERD (8 [IQR 0-17] vs 3 [IQR 0-10] [*P = .*027]).

LMS was associated with LoS in patients with hyposmia (OR = 2.66 [95% CI = 1.27-5.53] [*P = .*009]) but mainly in those with anosmia (OR = 16.67 [95% CI = 4.43-62.67] [*P* < .0001]) (area under the curve = 0.70 [95% CI = 0.62-0.77]).

Finally, LMS score showed a negative correlation with asthma control (determined according to Asthma Control Test score) in patients with CRSwNP (*r*= –0.249; *P = .*034) but not in patients with CRSsNP (Fig 3, *B*).

### Factors associated with severe asthma (multivariate analysis)

**Fig 4:**
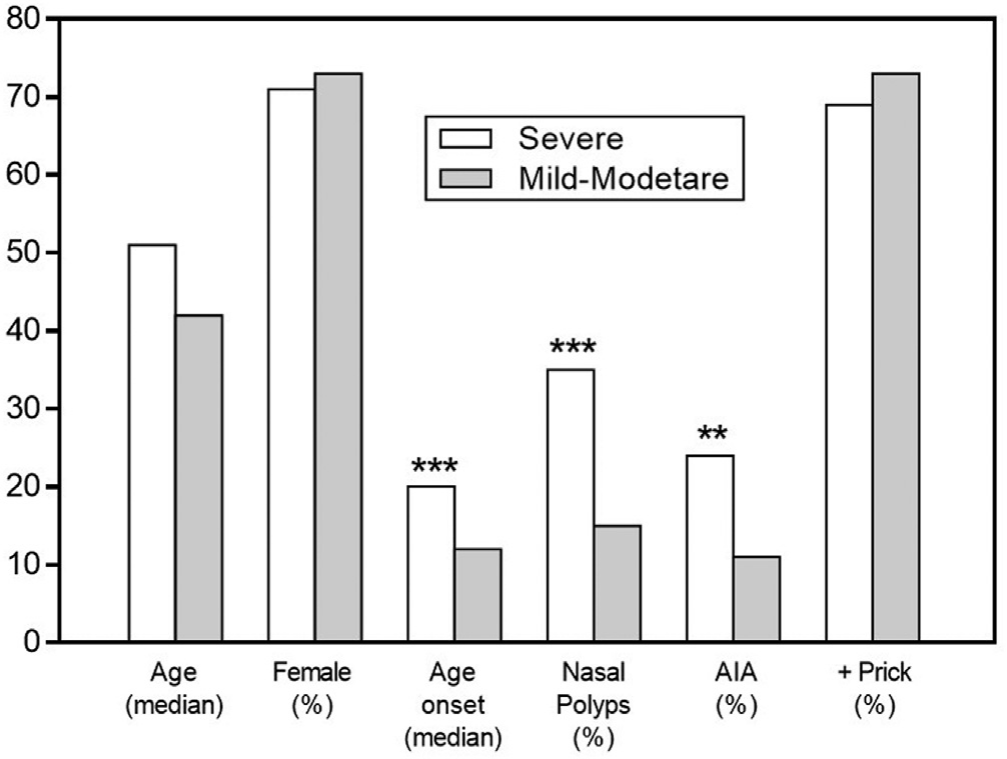
Age, sex, age of asthma onset, prevalence of nasal polyps, AIA, and atopy in patients with severe asthma and patients with mild-to-moderate persistent asthma. ∗∗*P* < .01; ∗∗∗*P* < .001.

As already mentioned, CRSwNP (OR = 3.4 [*P* < .001]) and AERD/N-ERD (OR = 7.8; [*P* < .001]) were associated with severe asthma, as were high symptom score, poor lung function (*P* < .001), need for intense treatment (use of oral corticosteroids) (*P* < .001), and exacerbation rate (*P* < .001). Patients with severe asthma were older (*P* < .001), had late-onset asthma (*P* < .001), and showed a higher sinus occupancy (*P* < .001), which was correlated with poor asthma control. Multiple logistic regression analysis showed that CRSwNP, AIA, and late-onset asthma were independently associated with severe asthma (Fig 4).

### Factors associated with severe and uncontrolled asthma

Multiple logistic analysis by univariate (see Table E2 in this article’s Online Repository at www.jaci-global.org) and multivariate regression analysis (Table II), showed that FEV_1_ (%) and use of oral corticosteroids were associated with both severe and uncontrolled asthma. CRSwNP, AERD/N-ERD, and late-onset asthma were independently associated with only severe asthma, and hyposmia was associated only with poor asthma control.

**Table II:** Factors associated with severe and uncontrolled asthma by backward multivariate regression analysis

Factor	Severe asthma (GINA)				Uncontrolled asthma (ACT score < 20)			
	Adjusted OR	95% CI		*P* value	Adjusted OR	95% CI		*P* value
Time since asthma onset	1.02	1.00	1.05	.022				
Sinonasal comorbidity				.001				
No	1							
NAR	4.98	0.65	38.12	.122				
AR	11.81	1.73	80.74	.012				
CRSsNP	8.07	1.15	56.49	.035				
CRSwNP	25.16	3.64	173.86	.001				
LoS								.014
Normosmia					1			
Hyposmia					2.29	1.17	4.46	.015
Anosmia					0.65	0.21	2.03	.457
FEV_1_ (%)	0.36	0.24	0.52	.000	0.69	0.49	0.97	.032
Oral steroid intake	14.56	5.67	37.38	.000	4.11	1.69	10.00	.002
AERD/N-ERD	2.13	1.04	4.36	.039				

*ACT*, Asthma Control Test.

## Discussion

To our knowledge, this is the first study to show the combined prevalence of rhinitis and CRS in patients with asthma by using a systematic approach based on definitions and classifications of the international consensus guidelines and their relationship with asthma severity and control, as well as to define them as different treatable traits in asthmatic patients.

The IRIS-Asthma study shows that CRSwNP is associated with severe asthma, whereas CRSwNP severity is associated with worse asthma control.

CRSwNP, together with AERD/N-ERD and late-onset asthma, appear as independent factors associated with severe asthma in contrast to mild-to-moderate asthma. Sinonasal occupancy (as indicated by LMS) and anosmia are associated with worse control, thus supporting the role of LoS as a clinical marker of severity.^33^^,^^34^

Our data also support a frequent late-onset asthma disease pattern^35^^,^^36^ in the intrinsic severe asthma subgroup, thus differentiating these patients from atopic patients with early-onset asthma.^10^ In consequence, there are 2 major sinonasal phenotypes or “traitable traits” in patients with asthma: AR, which is associated with mild-to-moderate asthma, and CRSwNP which is mainly associated with nonatopic severe asthma, reaffirming other studies.^37^, ^38^, ^39^, ^40^

The need for a better understanding of asthma heterogeneity prompted implementation of the ENFUMOSA^41^ study, which described the clinical characteristics of a cohort of asthmatic patients from several European countries, showing female sex, neutrophilic inflammation, and lower atopy as the key findings in severe asthma. Despite being a hallmark study regarding phenotyping of asthmatic patients, the ENFUMOSA study did not consider the evaluation of upper airways in patients with asthma. Similarly, Haldar et al^42^ proposed a mathematic cluster analysis to provide a framework for identifying distinct phenotypes, with specific pathophysiologic abnormalities predicting the therapeutic response and identifying the early-onset atopic and late-onset nonatopic asthma as 2 different phenotypes. That study also did not mention the impact of upper airway diseases (rhinitis or CRS) in the phenotyping of asthma. Since then, multiple approaches have been made to identify different types or phenotypes of patients with asthma with different characteristics and their evolution, management, and prognosis that need to be considered to precisely treat to target.

The relationship between severe asthma and CRS has been acknowledged, with more recent studies showing the relationship of severe asthma with CRSwNP.^6^^,^^43^^,^^44^

The present study, which is based on the upper airway multimorbidity in patients with asthma and their relationship with asthma severity and control, provides a useful method to phenotype asthma. Importantly, it finds that asthma associated with CRSwNP is the most severe type of asthma, conditioning its severity and control, which is clearly in alignment with the recent publication of Laidlaw et al^45^ identifying CRSwNP with comorbid AERD/N-ERD and asthma as a most severe and difficult-to-treat disease that may be the main target for type 2 biologic treatments.

Association of asthma with CRSwNP has a high clinical, social, and economic burden,^3^^,^^17^ especially in patients with AERD/N-ERD.^46^

Like the data of other studies^9^^,^^22^ reporting phenotypic characterization of population with severe asthma, our data fit the observation that patients with nonsevere asthma, patients with severe asthma are characterized by a higher proportion of negative results of SPTs to aeroallergens, higher prevalence of comorbid CRS, and late onset.^35^^,^^36^^,^^43^

In addition, patients with CRSwNP are more likely to have an altered microbiome in nasal mucosa colonization by *Staphylococcus aureus* and, through the accumulation of associated immune cells, tissue injury leading to loss of the mucosal barrier. *S aureus* enterotoxins act as superantigens and induce subsequent local production of polyclonal IgE that enhances mast cell degranulation contributing to chronic inflammation. In people with asthma, IgE to *S aureus* enterotoxins is associated with greater severity of asthma^47^^,^^48^ and serves to predict the severity and occurrence of asthma exacerbations. Functions of IgE in asthma may be similar to those in nasal polyposis.

In our study, we observed that CRSwNP occurs with similar frequency in patients with atopic asthma and patients with nonatopic asthma and that total IgE, which is correlated with eosinophil and type 2 biomarkers,^23^ is significantly higher in patients with severe asthma with CRSwNP independently of their allergic status. This finding agrees with previous findings regarding the role of IgE in patients with intrinsic asthma and patients with CRSwNP and the possible role of bacterial superantigens in the pathophysiology of patients with severe asthma and high IgE levels^47^^,^^48^ differing from that of patients with AR in terms of polyclonal IgE while indicating the importance of assessing upper airway disease when phenotyping asthma. This finding may also help to explain the effect of anti-IgE biologics (omalizumab)^49^ in patients with nonatopic asthma with CRSwNP.

Although a significant correlation between sinus occupancy (LMS) and symptom scores and asthma severity has been described,^6^^,^^50^ our study has interestingly found a relationship between poor asthma control and high LMS only in asthmatic patients with comorbid CRSwNP, which was not described previously.

In the present study, we found a prevalence of AERD/N-ERD of 15% among patients with asthma. In addition, AERD/N-ERD was clearly associated with severe asthma,^16^^,^^51^ high sinus occupancy in patients with CRSwNP, and greater LoS than in asthmatic patients with no AERD/N-ERD.^52^

The finding of anosmia being much more prevalent in patients with severe asthma supports the idea of this symptom serving as a clinical marker of severe asthma while reinforcing its assessment in daily clinical asthma practice. Overall, our results are consistent with those of other studies^44^ reporting that CRSwP, aspirin/NSAID sensitivity, and late onset of asthma are independent and crucial factors linked to severe asthma.

With regard to body mass index, in our study patients with severe asthma did not differ significantly from patients with nonsevere asthma, whereas other studies have associated severe asthma with obesity.^53^ Although the cohort had more female participants (70%), female sex was not related to asthma severity in our patients. This is in contrast with the findings of other studies previous.^41^^,^^54^

The strengths of our study are (1) the large sample of patients with asthma who have been included prospectively in this multicentric study that is strictly phenotyped for upper airway diseases; (2) the fact that this study reflects a real-life multidisciplinary approach with pulmonologists and ENT specialists, which implies a high external validity to be implemented in clinical practice; and (3) the fact that this study describes the existence of different “treatable traits” within a group of asthmatic patients with upper airway disease, either rhinitis or CRS, that may have an impact on asthma severity and control and hence need to be considered when diagnosing and treating patients with asthma.

On the other hand, the limitations of this multicenter prospective study include an information bias during recruitment that cannot be excluded as an important challenge to recruitment of patients with intermittent asthma in most participating centers.

In addition, the study results are not intended to reflect the real prevalence of the different levels of asthma severity, as its aim was to describe the association of upper airway multimorbidity in patients with asthma with different levels of severity.

In conclusion, our IRIS-Asthma study reports that compared with patients with mild-to-moderate persistent asthma, patients with severe asthma have a predominant nonatopic airway inflammation with CRSwNP, whereas sinus occupancy and anosmia, which are 2 main characteristics of CRSwNP, impair asthma control. Furthermore, the presence of CRSwNP, late-onset asthma, and AERD/N-ERD are crucial clinical features that are independently associated with severe asthma. Overall, the present findings support the relevance of evaluating the upper airway while giving high importance to an integral multidisciplinary approach in the management of multimorbid asthma and sinonasal diseases, either rhinitis or CRS, under the united airway disease umbrella. Future clinical guidelines should put additional effort into the presence of upper and lower airway multimorbidity as more common “treatable traits” to improve their management and follow-up.

## Disclosure statement

This study was designed and conducted by the Rhinitis, Rhinosinusitis and Polyposis Group of the Spanish SEPAR Asthma Area and partially funded by MSD, Spain and the Integral Research Program (PII) of SEPAR. The funders had no role in design and conduct of the study; collection, analysis, and interpretation of the data; or preparation, review, or approval of the article. The actual sponsor is the Integral Research Program (PII) of SEPAR.

Disclosure of potential conflict of interest: J. A. Castillo has received research grants from 10.13039/100004325AstraZeneca, Boehringer Ingelheim, GSK, MSD, and Uriach and attended speaker bureaus and/or advisory boards for ALK, AstraZeneca, GSK, Novartis, and Sanofi-Genzyme. In the last 3 years, V. Plaza has received honoraria from AstraZeneca, Boehringer Ingelheim, Chiesi, Gebro, GSK, and Sanofi for speaking at sponsored meetings; received assistance from AstraZeneca and Chiesi for travel to meetings; acted as consultant for AstraZeneca, GSK, and Sanofi; and received funding and/or grant support for research projects from a variety of government agencies and not-for-profit foundations, as well as from AstraZeneca, Chiesi, and Menarini. B. Juliá is a full-time employee of MSD. J. Mullol has received research grants, attended speaker bureaus and/or advisory boards, and received consulting fees from 10.13039/100004325AstraZeneca, 10.13039/100004328Genentech, Glenmark, GSK, Menarini, Mitsubishi-Tanabe Pharma, MSD, Noucor/Uriach Group, Novartis, Proctor & Gamble, 10.13039/100009857Regeneron Pharmaceuticals Inc, and Sanofi-Genzyme, UCB Pharma, and Viatris. The rest of the authors declare that they have no relevant conflicts of interest.

**Key messages**

• Asthma phenotyping should include upper airway evaluation and a multidisciplinary approach.
• AR and CRSwNP are identified as *treatable treats* in asthma: CRSwNP is associated with severe uncontrolled asthma, mainly nonatopic, whereas AR is associated to mild-to-moderate asthma.
• Anosmia and sinus occupancy in computed tomography are correlated with worse asthma control.

## Supplementary data

Supplementary Table E1

Supplementary Table E2
