# Supplementary material for: Chronic rhinosinusitis with nasal polyps and allergic rhinitis as different multimorbid treatable traits in asthma
Source: J Allergy Clin Immunol Glob. 2023 Jul 3;2(4):100134. doi: 10.1016/j.jacig.2023.100134 (PMC10510007; doi:10.1016/j.jacig.2023.100134)
Supplement: Supplementary Table E1 [file mmc1.docx]

| **Table E1.** Demographic, clinical and functional characteristics of asthma patients according to sinonasal multimorbidities. | | | | | | | | | | | | | | |
| --- | --- | --- | --- | --- | --- | --- | --- | --- | --- | --- | --- | --- | --- | --- |
| **Asthma characteristics** |  | **Global** | | **No sinonasal disease** | | **NAR** | | **AR** | | **CRS**  **without NP** | | **CRS**  **with NP** | | **P** |
| Sinonasal comorbidity ^2^ |  | 492 (100) | | 70 (14.2) | | 62 (12.6) | | 182 (37.0) | | 82 (16.7) | | 96 (19.5) | |  |
| Age, years^1^ |  | 46 | (33-58) | 52 | (38-63) | 55 | (36-61) | 43** | (31-52)^¤¤^ | 41*** | (30-52) | 50 | (38-58)^§§^ | <0.001 |
| Time since asthma onset, years^1^ |  | 13 | (5-25) | 13 | (4-30) | 10 | (3-18) | 14 | (6-23)^¤^ | 13 | (5-27) | 16 | (5-27) | 0.205 |
| BMI,^1^  kg/m^2^ |  | 26 | (23-30) | 28 | (24-31) | 26 | (23-31) | 27 | (23-30) | 26* | (23-29) | 26* | (23-29) | 0.059 |
| Gender, female^2^ |  | 347 | (70.5) | 51 | (72.9) | 50 | (80.6) | 122 | (67.0)^¤^ | 63 | (76.8) | 61 | (63.5)^§^ | 0.087 |
| Asthma severity^2^ | Intermitent | 86 | (17.5) | 10 | (14.3) | 14 | (22.6) | 35 | (19.2) | 16 | (19.5) | 11 | (11.5) | <0.001 |
|  | Mild Persistent | 121 | (24.6) | 24 | (34.3) | 17 | (27.4) | 45 | (24.7) | 24 | (29.3) | 11 | (11.5) |  |
|  | Moderate Persistent | 154 | (31.3) | 21 | (30.0) | 21 | (33.9) | 62 | (34.1) | 22 | (26.8) | 28 | (29.2) |  |
|  | Severe Persistent | 131 | (26.6) | 15 | (21.4) | 10 | (16.1) | 40 | (22.0) | 20 | (24.4) | 46*** | (47.9)^§§^ |  |
| ACT^1^ |  | 21 | (16-24) | 21 | (18-25) | 22 | (18-24) | 21 | (17-24) | 20 | (15-23) | 20 | (16-24) | 0.144 |
| Asthma exacerbations^2^  (last three months)^2^ | None | 310 | (63.0) | 50 | (71.4) | 38 | (61.3) | 123 | (67.6) | 50 | (61.0) | 49 | (51.0) | 0.498 |
|  | From 1 to 3 | 165 | (33.5) | 17 | (24.3) | 23 | (37.1) | 54 | (29.7) | 29 | (35.4) | 42 | (43.8) |  |
|  | More than 4 | 17 | (3.5) | 3 | (4.3) | 1 | (1.6) | 5 | (2.7) | 3 | (3.6) | 5 | (5.2) |  |
| LMS (by CT scan)^1^ |  | 4 | (0-11) | 1 | (0-6) | 0 | (0-0) | 0 | (0-0) | 5 | (1-7) | 10 | (6-18)^§§§^ | <0.001 |
| Loss of smell, VAS ^2^ | Normosmia | 167 | (43.6) | 23 | (95.8) | 28 | (58.3) | 74 | (55.6) | 26 | (31.7) | 16 | (16.7) | <0.001 |
|  | Hyposmia (10-70 mm) | 159 | (41.5) | 1 | (4.2) | 18 | (37.5) | 54 | (40.6) | 45 | (54.9) | 41 | (42.7) |  |
|  | Anosmia (>70 mm) | 57 | (14.9) | 0 | (0.0) | 2** | (4.2) | 5** | (3.8) | 11*** | (13.4) | 39*** | (40.6)^§§§^ |  |
| Blood eosinophils (cell / | μL),^1^ | 250 | (112-415) | 292 | (160-592) | 291 | (105-400) | 200 | (100-320) | 289 | (180-453) | 310 | (200-595) | 0.024 |
| FeNO, ppb^1^ |  | 26 | (15-45) | 26 | (15-49) | 22 | (11-31) | 22 | (14-40) | 29 | (13-45) | 39 | (23-65) | 0.001 |
| FEV_1 ,_%^1^ |  | 91 | (75-103) | 93 | (77-108) | 93 | (78-107) | 93 | (77-103) | 88 | (73-100) | 82 | (67-95) | 0.013 |
| IgE, IU/mL^1^ |  | 150 | (56-377) | 110 | (60-230) | 53* | (16-151) | 161* | (73-416)^¤¤¤^ | 176 | (60-348) | 184* | (99-547) | 0.001 |
| Oral steroid intake^2^ |  | 55 | (11.2) | 6 | (8.6) | 6 | (9.7) | 13 | (7.1) | 10 | (12.2) | 20* | (20.8) | 0.013 |
| Positive skin prick test^2^ |  | 298 | (71.1) | 34 | (64.2) | 0* | (0.0) | 162*** | (100.0) | 43 | (76.8) | 59 | (67.8) | <0.001 |
| AERD/N-ERD^2^ |  | 72 | (15.2) | 3 | (4.5) | 11 | (18.6) | 16 | (9.3)^¤^ | 14* | (17.5) | 28*** | (29.8)^§^ | <0.001 |
| Smoking habit^2^ |  | 47 | (9.6) | 10 | (14.3) | 6.0 | (9.7) | 19 | (10.4) | 9 | (11.0) | 3** | (3.1)^§^ | 0.149 |
| Packs per year^1^ |  | 5 | (2-20) | 30 | (15-30) | 9 | (5-13) | 2** | (1-15) | 6 | (3-31) | 2* | (1-5) | 0.049 |
| ^1^ median (25-75 interquartil range)  ^2^ n (%)  Each phenotype vs. without sinonasal disease: *p <0.05; **p<0.01; *** p<0.001  AR vs NAR: ^¤^p <0.05;^¤¤^p<0.01;^¤¤¤^p<0.001  CRSwNP vs CRSsNP: ^§^p <0.05; ^§§^p<0.01;^§§§^p<0.001 | | | | | | | | | | | | | | |

ACT, Asthma Control Test; BMI, Body Mass Index; NAR, non-allergic rhinitis; AR; allergic rhinitis; CRSsNP, CRS without nasal polyps; CRSsNP, CRS with nasal polyps, CT, computed tomography; LMS (Lund-McKay score), FeNO, exhaled nitric oxide; Aspirin or NSAID-exacerbated respiratory disease (AERD/N-ERD)
