# Supplementary material for: Chronic rhinosinusitis with nasal polyps and allergic rhinitis as different multimorbid treatable traits in asthma
Source: J Allergy Clin Immunol Glob. 2023 Jul 3;2(4):100134. doi: 10.1016/j.jacig.2023.100134 (PMC10510007; doi:10.1016/j.jacig.2023.100134)
Supplement: Supplementary Table E2 [file mmc2.docx]

Table E2. Factors associated to severe and uncontrolled asthma by univariate regression analysis.

|  | **Severe Asthma**  **(GINA)** | | | | **Uncontrolled Asthma (ACT<20)** | | | |
| --- | --- | --- | --- | --- | --- | --- | --- | --- |
|  | Univariate Odds Ratio | 95% CI | |  | Univariate Odds Ratio | 95% CI | |  |
|  |  | Lower | Upper | p |  | Lower | Upper | p |
| Gender, female^2^ | 1.03 | 0.66 | 1.60 | 0.8920 | 1.29 | 0.86 | 1.92 | 0.2170 |
| Time since asthma onset, years^1^ | 1.03 | 1.02 | 1.05 | 0.0001 | 1.01 | 0.99 | 1.02 | 0.4420 |
| BMI, ^1^ kg/m^2^ | 1.05 | 1.01 | 1.09 | 0.0190 | 1.01 | 0.98 | 1.05 | 0.5230 |
| Sinonasal comorbidity ^2^ |  |  |  | 0.0001 |  |  |  | 0.2950 |
| No | 1 |  |  |  | 1 |  |  |  |
| NAR | 0.71 | 0.29 | 1.71 | 0.4390 | 1.03 | 0.50 | 2.13 | 0.9270 |
| AR | 1.03 | 0.53 | 2.02 | 0.9250 | 1.20 | 0.67 | 2.14 | 0.5370 |
| CRSsNP | 1.18 | 0.55 | 2.53 | 0.6660 | 1.62 | 0.84 | 3.14 | 0.1530 |
| CRSwNP | 3.37 | 1.68 | 6.78 | 0.0010 | 1.72 | 0.91 | 3.26 | 0.0960 |
| LMS (by CT scan)^1^ | 1.06 | 1.02 | 1.11 | 0.0060 | 1.04 | 1.00 | 1.08 | 0.0760 |
| Loss of smell (VAS)^2^ |  |  |  | 0.0001 |  |  |  | 0.1340 |
| Normosmia | 1 |  |  |  | 1 |  |  |  |
| Hyposmia | 1.72 | 1.03 | 2.88 | 0.0400 | 1.55 | 0.99 | 2.41 | 0.0530 |
| Anosmia | 4.07 | 2.13 | 7.78 | 0.0001 | 1.06 | 0.57 | 1.98 | 0.8430 |
| Blood eosinophilia,(cell/ μL) ^1^ | 1.00 | 0.99 | 1.01 | 0.8440 | 0.98 | 0.95 | 1.00 | 0.0900 |
| FeNO, ppb ^1^ | 1.00 | 0.99 | 1.01 | 0.7930 | 1.00 | 1.00 | 1.01 | 0.2910 |
| FEV_1 ,_%^1^ | 0.27 | 0.20 | 0.37 | 0.0001 | 0.59 | 0.48 | 0.72 | 0.0001 |
| IgE, IU/mL ^1^ | 1.07 | 1.02 | 1.11 | 0.0030 | 1.05 | 1.01 | 1.09 | 0.0190 |
| Oral steroid intake^2^ | 21.17 | 9.97 | 44.93 | 0.0001 | 6.54 | 3.27 | 13.06 | 0.0001 |
| Positive skin prick test ^2^ | 0.87 | 0.54 | 1.38 | 0.5470 | 0.89 | 0.57 | 1.37 | 0.5910 |
| AERD/N-ERD^2^ | 2.28 | 1.36 | 3.82 | 0.0020 | 1.07 | 0.64 | 1.78 | 0.7950 |
| Smoking Habit^2^ | 0.23 | 0.08 | 0.66 | 0.0060 | 1.09 | 0.59 | 2.02 | 0.7720 |
| Packs per year^1^ | 1.00 | 0.91 | 1.09 | 0.9450 | 0.97 | 0.91 | 1.03 | 0.2820 |

ACT, Asthma Control Test; BMI, Body Mass Index; NAR, non-allergic rhinitis; AR; allergic rhinitis; CRSsNP, CRS without nasal polyps; CRSwNP, CRS with nasal polyps; LMS, Lund-MacKay score; CT, computed tomography; FeNO, exhaled nitric oxide; Aspirin or NSAID-exacerbated respiratory disease (AERD/N-ERD)
